# A rare case of pityriasis amiantacea of the scalp posing differential diagnosis challenges to fungal skin infections

**DOI:** 10.1186/1471-2334-13-S1-P74

**Published:** 2013-12-16

**Authors:** Simona-Roxana Georgescu, Vasile Benea, Mircea Tampa, Anca Malin-Benea, Alice Rusu

**Affiliations:** 1 Carol Davila University of Medicine and Pharmacy, Bucharest, Romania; 2 Clinical Hospital of Infectious and Tropical Diseases “Dr. Victor Babeş”, Bucharest, Romania

## Background

Pityriasis amiantacea is an uncommon clinical syndrome of the scalp with a particular reaction pattern seen in a number of inflammatory and infectious dermatoses such as seborrheic dermatitis, psoriasis, atopic dermatitis, contact dermatitis, lichen planus, pityriasis rubra pilaris and various superficial pyogenic or fungal infections. The syndromic diagnosis may be established by the infectionist or dermatologist by clinical examination; additional tests like histopathologic examination, bacteriologic, mycological exam and cultures are needed in order to diagnose the underlying disease, which in many cases remains unknown. The condition is characterized by thick, silvery, asbestos-like scales binding proximally the hair shafts. It is more common in women and in young patients. Common complications include temporary or scarring alopecia. The etiopathogeny, the methods of diagnosis as well as the management options are still being subjects for debate.

## Case report

We report the case of a 59 year-old female patient who addressed our clinic for a papulo-squamous condition that presented with massive adherent scales on the scalp. The local examination revealed a well circumscribed, slightly infiltrated pruritic patch, with an erythematous border and on its surface with numerous shiny, white clusters of thick scales. The scales were micaceous, adherent to the scalp and encircled bundles of hair giving a roof tiles appearance. The lesions had appeared about 7 months prior to the presentation, but no treatment was effective. The medical history revealed type II diabetes. The patient was under chronic antidiabetic medication and was otherwise healthy.

Laboratory tests were within normal range with the exception of glycemia - 168 mg/dL. The clinical diagnosis was of pityriasis (pseudotinea) amiantacea. In order to discover a possible underlying primary dermatosis (most often fungal skin infection - tinea capitis, psoriasis, seborrheic or atopic dermatitis) we proposed various tests, but the patient refused. The treatment consisted of topical keratolytic, antifungal and corticosteroid agents and systemic antifungal therapy. At the three weeks follow-up the abundant scales encasing tufts of hair had disappeared, leaving a slightly erythematous patch with fine scales on its surface. The patient is still under treatment and is being closely followed-up.

## Conclusion

Pityriasis amiantacea is a rarely encountered condition. In this case, the age of onset is unusual, as the condition mostly affects children. Recognizing the condition is of a paramount importance to the infectionist, as fungal infection of the skin (tinea capitis) is the main differential diagnosis to be considered.

